# Conformational dimorphism of 2,2′-methyl­enebis(isoindoline-1,3-dione)

**DOI:** 10.1107/S2056989018017425

**Published:** 2019-01-01

**Authors:** Tze Shyang Chia, Huey Chong Kwong, Ai Jia Sim, Weng Zhun Ng, Qin Ai Wong, C. S. Chidan Kumar, Ching Kheng Quah, Md. Azharul Arafath

**Affiliations:** a X-ray Crystallography Unit, School of Physics, Universiti Sains Malaysia, 11800 USM, Penang, Malaysia; b School of Chemical Sciences, Universiti Sains Malaysia, 11800 USM, Penang, Malaysia; c Department of Engineering Chemistry, Vidya Vikas Institute of Engineering and Technology, Visvesvaraya Technological University, Alanahalli, Mysuru 570 028, India; d Department of Chemistry, Shahjalal University of Science and Technology, Sylhet, 3114, Bangladesh

**Keywords:** crystal structure, polymorphism, isoindoline-1,3-dione, Hirshfeld surface analysis, PIXEL

## Abstract

In this study, a new monoclinic polymorph (space group *C*2/*c*) of 2,2′-methyl­enebis(isoindoline-1,3-dione), C_17_H_10_N_2_O_4_, is reported and compared to the previously reported triclinic polymorph (space group *P* ). Similarly, both polymorphs consist of a unique mol­ecule in the asymmetric unit (*Z*′ = 1). The mol­ecular conformations of the two polymorphs are very similar, as shown by the r.m.s. deviation of 0.368 Å (excluding all H atoms). The inter­molecular inter­actions of both polymorphs are described along with the Hirshfeld surface analysis, and the lattice energies are calculated.

## Abstract 2

A new monoclinic polymorph of 2,2′-methyl­enebis(isoindoline-1,3-dione) with *Z*′ = 1 is reported.

## Chemical context   

Phthalimide (or isoindoline-1,3-dione) derivatives with five-membered *N*-heterocycles have been proven to exhibit significant biological and pharmaceutical activities, and have also been used as dyes and heat-resistant polymers in industry (Chidan Kumar *et al.*, 2015 ▸; Then *et al.*, 2018 ▸). The first reported crystal structure of 2,2′-methyl­enebis(isoindoline-1,3-dione) (1*α*; Jiang *et al.*, 2007 ▸) crystallizes in the centrosymmetric triclinic space group *P* 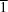 [*a* = 7.6660 (9) Å, *b* = 9.5810 (8) Å, *c* = 10.2780 (6) Å, *α* = 104.325 (3)°, *β* = 99.768 (4)°, *γ* = 96.030 (3)°, *Z* = 2, *Z*′ = 1 and *V* = 712.23 (11) Å^3^; Cambridge Structural Database (CSD; Groom *et al.*, 2016 ▸) refcode SINDID]. In this article, we report the second polymorphic form (1*β*) of 2,2′-methyl­enebis(isoindoline-1,3-dione) with *Z*′ = 1 and compare its properties with those of 1*α*. According to the Online Dictionary of Crystallography, polymorphism is the phenomenon in which the same chemical compound exhibits different crystal structures (IUCr, 2018 ▸).
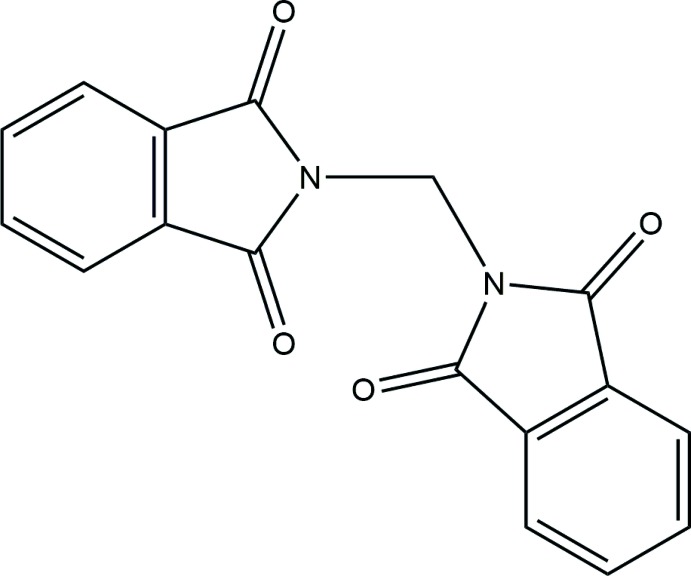


## Structural commentary   

**Figure 1:**
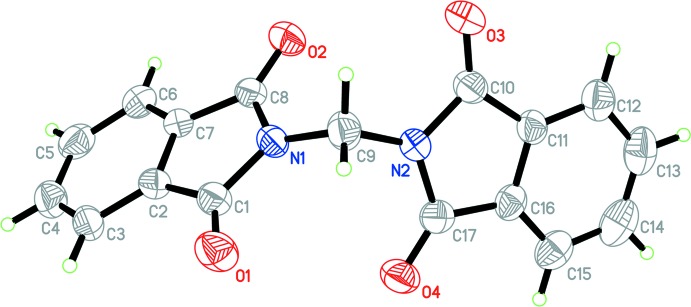
Mol­ecular structure of 1*β* with atom labels and 30% probability displacement ellipsoids.

**Figure 2:**
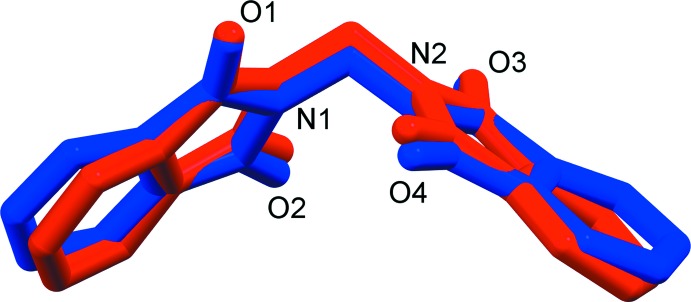
An overlay diagram for the mol­ecules of 1*α* (red) and 1*β* (blue).

The asymmetric units of polymorphs 1*α* and 1*β* (Fig. 1 ▸) each contain a unique mol­ecule of 2,2′-methyl­enebis(isoindoline-1,3-dione), which consists of two phthalimide groups and a methyl­ene bridge. The phthalimide groups are nearly planar with maximum deviations of 0.029 (1) and 0.059 (1) Å for 1*α*, and 0.040 (4) and 0.064 (3) Å for 1*β*. There are two degrees of freedom to characterize the mol­ecular conformations of 1*α* and 1*β*: these are the torsion angles C1—N1—C9—N2 [106.7 (1) and 117.4 (3)°, respectively] and N1—C9—N2—C10 [109.2 (1) and 117.6 (3)°, respectively]. Generally, the mol­ecule of 1*β* deviates only slightly from that of 1*α*, as indicated by a r.m.s. deviation of 0.368 Å (excluding all H atoms) (Fig. 2 ▸). The mean planes of the phthalimide rings for 1*β* make a dihedral angle of 76.12 (8)°, which is smaller than that of 88.96 (4)° observed in polymorph 1*α*. The calculated density and Kitaigorodskii packing index (Spek, 2009 ▸) for 1*β* (1.469 Mg m^−3^ and 70.0%) are slightly higher than those observed for 1*α* (1.428 Mg m^−3^ and 69.0%).

## Supra­molecular features   

**Figure 3:**
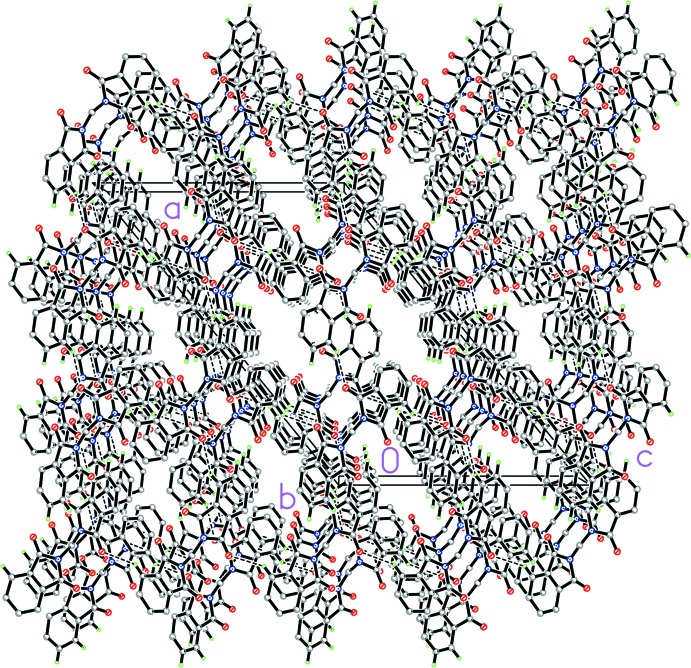
A partial crystal packing diagram of 1*β* viewed along the *b* axis. Dashed lines represent weak inter­molecular C—H⋯O hydrogen bonds. Hydrogen atoms which are not involved in hydrogen bonding are omitted for clarity.

The crystal packing of 1*α* features weak inter­molecular C—H⋯O hydrogen bonds and π–π inter­actions between neighboring phthalimide units. In the crystal structure of 1*β* (Fig. 3 ▸), the mol­ecules are connected by weak inter­molecular C—H⋯O hydrogen bonds (Table 1 ▸), forming a three-dimensional network. The crystal structure of 1*β* also features weak π–π inter­actions between two C2–C7 phenyl rings (symmetry code: −*x*, −*y* + 1, −*z*) and between N1/C1/C2/C7/C8 and C11–C16 rings (symmetry codes: −*x* + 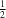, *y* + 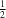, −*z* + 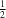 and −*x* + 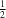, *y* − 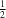, −*z* + 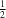), with centroid-to-centroid distances of 3.664 (3) and 3.938 (3) Å, respectively.

## Hirshfeld surface analysis   

**Figure 4:**
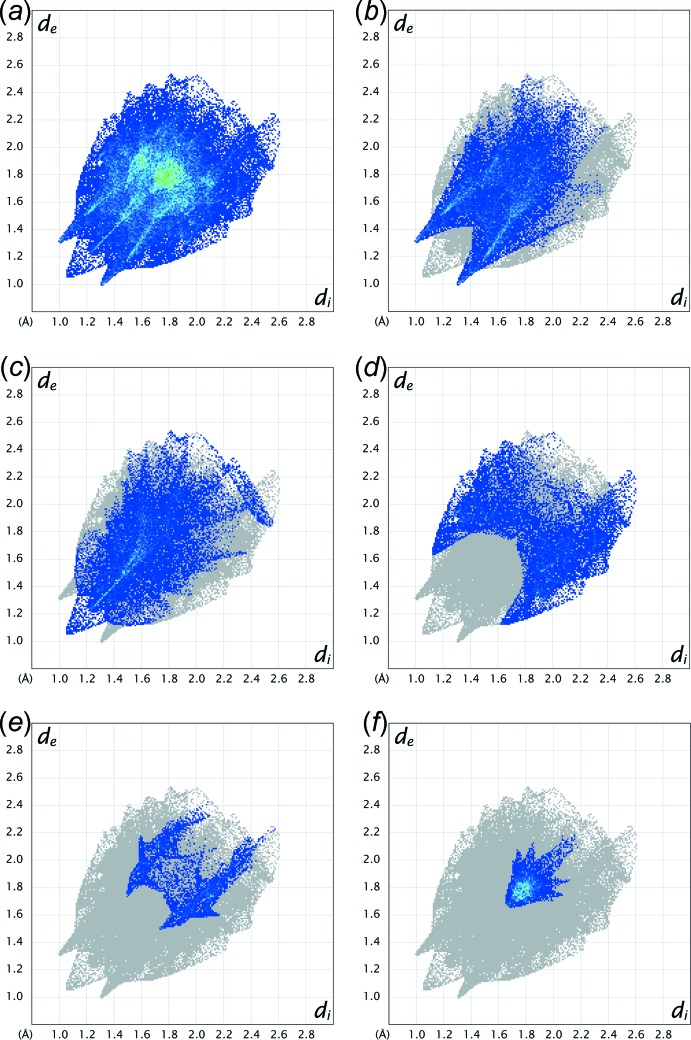
The two-dimensional fingerprint plots of 1*β* for (*a*) all, (*b*) H⋯O/O⋯H, (*c*) H⋯H, (*d*) H⋯C/C⋯H, (*e*) O⋯C/C⋯O and (*f*) C⋯C contacts. *d*_i_ and *d*_e_ are the distances from the Hirshfeld surface to the nearest atom inter­ior and exterior, respectively, to the surface.

**Figure 5:**
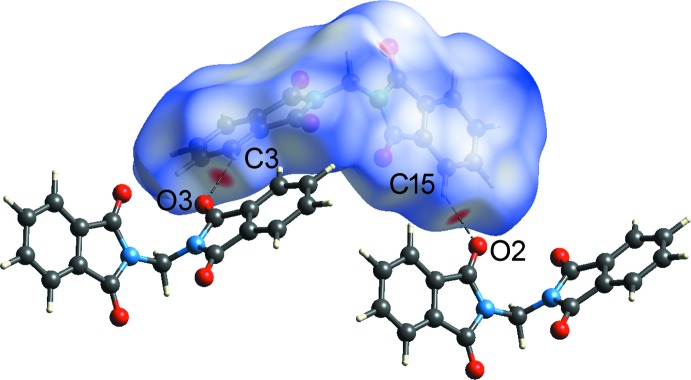
The Hirshfeld surface mapped over *d*_norm_ for the mol­ecule in the asymmetric unit of 1*β* hydrogen-bonded to two neighbouring mol­ecules.

**Figure 6:**
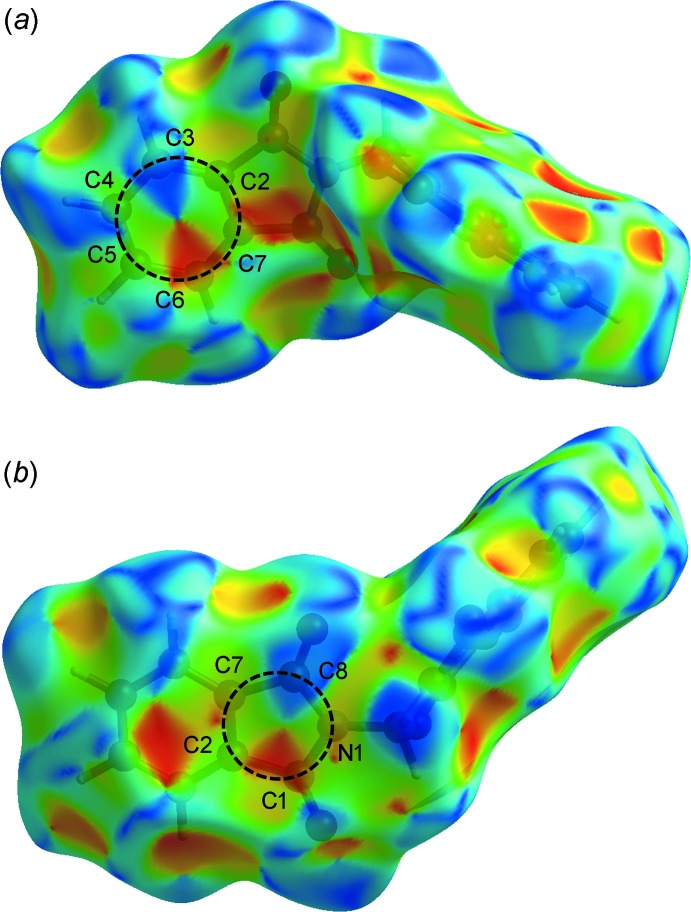
The Hirshfeld surface mapped over shape-index for 1*β*.

**Figure 7:**
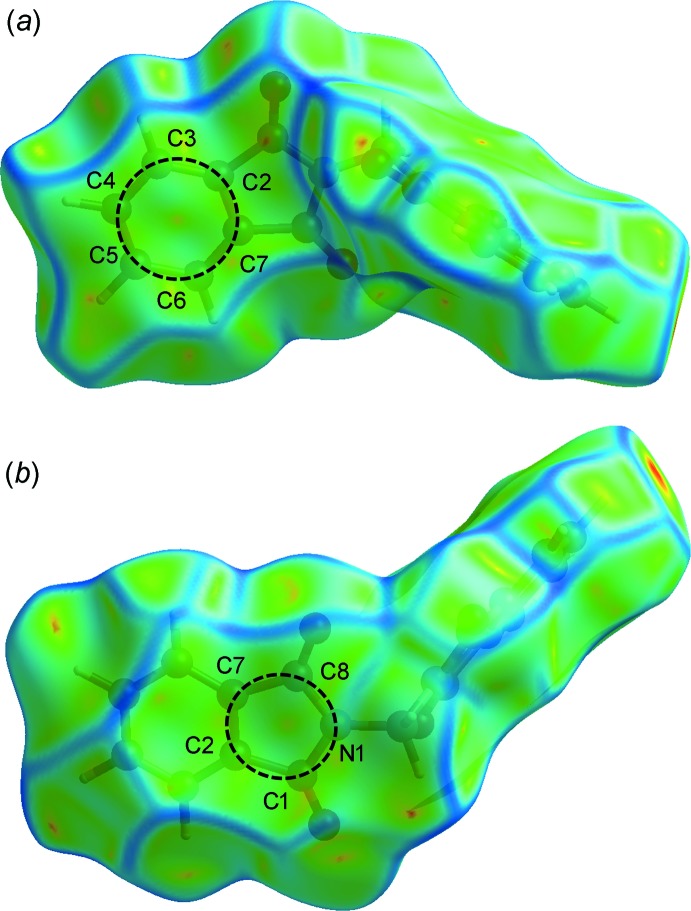
The Hirshfeld surface mapped over curvedness for 1*β*.

The Hirshfeld surface analysis and two-dimensional fingerprint plots were performed using *CrystalExplorer* version 17.5 (Spackman & Jayatilaka, 2009 ▸; Spackman & McKinnon, 2002 ▸; Turner *et al.*, 2017 ▸). The H⋯O/O⋯H contact is the most populated contact and contributes 38.2 and 34.4% of the total inter­molecular contacts of 1*α* and 1*β* (Fig. 4 ▸), respectively. The large red spots on the Hirshfeld surface mapped over *d*_norm_ for 1*β* (Fig. 5 ▸) correspond to the inter­molecular C3—H3*A*⋯O3 and C15—H15*A*⋯O2 hydrogen-bonds. The tips of the pseudo-mirrored sharp spikes at *d*_e_ + *d*_i_ ≃ 2.32 Å represent the shortest H⋯O/O⋯H contacts, corresponding to the inter­molecular C3—H3*A*⋯O3 hydrogen-bond. The H⋯H contact is the second most populated contact and contributes 25.4 and 26.5% of the total inter­molecular contacts of 1*α* and 1*β*, respectively. The shortest H⋯H contacts of 1*α* (symmetry code: −*x*, −*y*, −*z* + 1) and 1*β* (symmetry code: −*x*, *y*, −*z* − 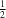) are illustrated as two sharp tips along the diagonal of their two-dimensional fingerprint plots at *de* ≃ *di* ≃ 1.06 and 1.21 Å [Fig. 4 ▸(*c*)], respectively. The percentages of contribution of H⋯C/C⋯H, O⋯C/C⋯O and C⋯C contacts to the Hirshfeld surface are 20.6, 3.3 and 8.9%, respectively, for 1*α*, and 20.8, 7.9 and 6.7%, respectively, for 1*β* (Fig. 4 ▸). The absence of significant C—H⋯π inter­actions in the crystal structure of 1*β* is indicated by the absence of characteristic ‘wings’ in the fingerprint plot of H⋯C/C⋯H contacts [Fig. 4 ▸(*d*)]. The C⋯C contacts appear as a unique ‘triangle’ focused at *d*_e_ ≃ *d*_i_ ≃ 1.75 Å [Fig. 4 ▸(*f*)]. The inter­molecular π–π inter­actions are illustrated as unique patterns of red and blue ‘triangles’ on the shape-index surface (Fig. 6 ▸), and flat regions on the curvedness surface (Fig. 7 ▸), of the C2–C7, N1/C1/C2/C7/C8 and C11–C16 rings.

## Lattice energy calculation   

The C—H bond lengths in 1*α* and 1*β* were normalized to 1.08 Å and the lattice energies were calculated by using the *CLP-PIXEL* software package (Gavezzotti, 2003 ▸, 2008 ▸). The calculated lattice energy of 1*α* (130.3 kJ mol^−1^) is slightly larger than for 1*β* (128.5 kJ mol^−1^), indicating that 1*α* is slightly more stable than 1*β* under ambient conditions.

## Synthesis and crystallization   

Single crystals of 1*β* were obtained from an unsuccessful synthesis of 2-{[(3-iodo­pyridin-4-yl)amino]­meth­yl}isoindoline-1,3-dione by reacting *N*-(bromo­meth­yl)phthalimide (1 mmol) and 4-amino-3-iodo­pyridine (1 mmol) in *N*,*N*-di­methyl­formamide (8 ml) with the presence of a catalytic amount of anhydrous potassium carbonate. The reaction solution was stirred for about 2 h at room temperature. Once the reaction was complete, the resultant mixture was poured into a beaker of ice-cooled water to obtain a precipitate (Then *et al.*, 2018 ▸), which was then filtered, washed with distilled water and dried. Crystals suitable for X-ray analysis were obtained by slow evaporation of a methanol solution.

## Refinement   

Crystal data, data collection and structure refinement details of 1*β* are summarized in Table 2 ▸. All H atoms were positioned geometrically (C—H = 0.93 and 0.97 Å) and refined using a riding model, with *U*_iso_(H) = 1.2*U*_eq_(C).

## Supplementary Material

Crystal structure: contains datablock(s) I. DOI: 10.1107/S2056989018017425/jj2205sup1.cif

Structure factors: contains datablock(s) I. DOI: 10.1107/S2056989018017425/jj2205Isup2.hkl

Supporting information file. DOI: 10.1107/S2056989018017425/jj2205Isup3.cml

CCDC reference: 1884044

Additional supporting information:  crystallographic information; 3D view; checkCIF report

## Figures and Tables

**Table 1:** Hydrogen-bond geometry (Å, °)

*D*—H⋯*A*	*D*—H	H⋯*A*	*D*⋯*A*	*D*—H⋯*A*
C3—H3*A*⋯O3^i^	0.93	2.43	3.150 (6)	134
C4—H4*A*⋯O4^ii^	0.93	2.60	3.300 (5)	133
C15—H15*A*⋯O2^iii^	0.93	2.46	3.271 (7)	146

Symmetry codes: (i) 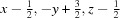; (ii) 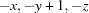; (iii) 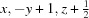.

**Table 2:** Experimental details

Crystal data	
Chemical formula	C_17_H_10_N_2_O_4_
*M*_r_	306.27
Crystal system, space group	Monoclinic, *C*2/*c*
Temperature (K)	296
*a*, *b*, *c* (Å)	26.296 (5), 7.9996 (15), 16.987 (4)
β (°)	129.165 (10)
*V* (Å^3^)	2770.5 (10)
*Z*	8
Radiation type	Mo *K*α
μ (mm^−1^)	0.11
Crystal size (mm)	0.44 × 0.13 × 0.02

Data collection	
Diffractometer	Bruker SMART APEXII DUO CCD area-detector
Absorption correction	Multi-scan (*SADABS*; Bruker, 2009 ▸)
*T*_min_, *T*_max_	0.649, 0.745
No. of measured, independent and observed [*I* > 2σ(*I*)] reflections	29675, 2444, 1213
*R*_int_	0.128
(sin θ/λ)_max_ (Å^−1^)	0.595

Refinement	
*R*[*F* ^2^ > 2σ(*F* ^2^)], *wR*(*F* ^2^), *S*	0.063, 0.158, 1.02
No. of reflections	2444
No. of parameters	208
H-atom treatment	H-atom parameters constrained
Δρ_max_, Δρ_min_ (e Å^−3^)	0.14, −0.17

Computer programs: *APEX2* and *SAINT* (Bruker, 2009 ▸), *SHELXS2013* and *SHELXTL* (Sheldrick, 2008 ▸), *SHELXL2014* (Sheldrick, 2015 ▸), *Mercury* (Macrae *et al.*, 2008 ▸), *PLATON* (Spek, 2009 ▸) and *publCIF* (Westrip, 2010 ▸).

## supplementary crystallographic information

### Crystal data

**Table:**

C_17_H_10_N_2_O_4_	*F*(000) = 1264
*M**_r_* = 306.27	*D*_x_ = 1.469 Mg m^−^^3^
Monoclinic, *C*2/*c*	Mo *K*α radiation, λ = 0.71073 Å
*a* = 26.296 (5) Å	Cell parameters from 1601 reflections
*b* = 7.9996 (15) Å	θ = 2.4–26.4°
*c* = 16.987 (4) Å	µ = 0.11 mm^−^^1^
β = 129.165 (10)°	*T* = 296 K
*V* = 2770.5 (10) Å^3^	Block, colourless
*Z* = 8	0.44 × 0.13 × 0.02 mm

### Data collection

**Table:**

Bruker SMART APEXII DUO CCD area-detector diffractometer	2444 independent reflections
Radiation source: fine-focus sealed tube	1213 reflections with *I* > 2σ(*I*)
Graphite monochromator	*R*_int_ = 0.128
φ and ω scans	θ_max_ = 25.0°, θ_min_ = 2.0°
Absorption correction: multi-scan (*SADABS*; Bruker, 2009)	*h* = −31→31
*T*_min_ = 0.649, *T*_max_ = 0.745	*k* = −9→9
29675 measured reflections	*l* = −20→20

### Refinement

**Table:**

Refinement on *F*^2^	0 restraints
Least-squares matrix: full	Hydrogen site location: inferred from neighbouring sites
*R*[*F*^2^ > 2σ(*F*^2^)] = 0.063	H-atom parameters constrained
*wR*(*F*^2^) = 0.158	*w* = 1/[σ^2^(*F*_o_^2^) + (0.0624*P*)^2^ + 0.5204*P*] where *P* = (*F*_o_^2^ + 2*F*_c_^2^)/3
*S* = 1.02	(Δ/σ)_max_ < 0.001
2444 reflections	Δρ_max_ = 0.14 e Å^−^^3^
208 parameters	Δρ_min_ = −0.17 e Å^−^^3^

### Special details

**Table:**

Geometry. All esds (except the esd in the dihedral angle between two l.s. planes) are estimated using the full covariance matrix. The cell esds are taken into account individually in the estimation of esds in distances, angles and torsion angles; correlations between esds in cell parameters are only used when they are defined by crystal symmetry. An approximate (isotropic) treatment of cell esds is used for estimating esds involving l.s. planes.

### Fractional atomic coordinates and isotropic or equivalent isotropic displacement parameters (Å^2^)

**Table:**

	*x*	*y*	*z*	*U*_iso_*/*U*_eq_	
O1	0.05520 (11)	0.9015 (3)	0.12678 (18)	0.0786 (8)	
O2	0.20385 (12)	0.6374 (3)	0.11771 (17)	0.0769 (8)	
O3	0.32974 (11)	0.7796 (3)	0.34998 (18)	0.0817 (8)	
O4	0.15774 (12)	0.6563 (3)	0.34093 (18)	0.0765 (8)	
N1	0.14191 (12)	0.7785 (3)	0.14765 (19)	0.0563 (8)	
N2	0.23369 (12)	0.7434 (3)	0.32431 (19)	0.0577 (8)	
C1	0.07580 (16)	0.8101 (4)	0.0966 (3)	0.0595 (9)	
C2	0.04023 (16)	0.7104 (4)	0.0028 (2)	0.0547 (9)	
C3	−0.02562 (16)	0.6944 (4)	−0.0726 (2)	0.0620 (10)	
H3A	−0.0554	0.7553	−0.0718	0.074*	
C4	−0.04608 (18)	0.5854 (5)	−0.1492 (3)	0.0777 (11)	
H4A	−0.0908	0.5706	−0.2014	0.093*	
C5	−0.0021 (2)	0.4972 (5)	−0.1507 (3)	0.0798 (12)	
H5A	−0.0176	0.4222	−0.2033	0.096*	
C6	0.06535 (19)	0.5173 (5)	−0.0753 (3)	0.0697 (10)	
H6A	0.0955	0.4600	−0.0768	0.084*	
C7	0.08469 (15)	0.6259 (4)	0.0011 (2)	0.0531 (9)	
C8	0.15090 (17)	0.6749 (4)	0.0923 (2)	0.0568 (9)	
C9	0.19381 (15)	0.8576 (5)	0.2405 (2)	0.0671 (10)	
H9A	0.1752	0.9399	0.2578	0.081*	
H9B	0.2216	0.9165	0.2305	0.081*	
C10	0.30011 (17)	0.7197 (5)	0.3748 (3)	0.0625 (10)	
C11	0.32396 (17)	0.6090 (4)	0.4617 (2)	0.0591 (9)	
C12	0.38561 (19)	0.5506 (5)	0.5381 (3)	0.0765 (11)	
H12A	0.4214	0.5788	0.5418	0.092*	
C13	0.3923 (2)	0.4479 (6)	0.6095 (3)	0.0930 (14)	
H13A	0.4336	0.4071	0.6628	0.112*	
C14	0.3398 (3)	0.4050 (5)	0.6036 (3)	0.0956 (14)	
H14A	0.3458	0.3341	0.6522	0.115*	
C15	0.2779 (2)	0.4654 (5)	0.5263 (3)	0.0784 (11)	
H15A	0.2420	0.4371	0.5222	0.094*	
C16	0.27123 (18)	0.5674 (4)	0.4564 (3)	0.0607 (9)	
C17	0.21289 (18)	0.6552 (4)	0.3696 (3)	0.0590 (9)	

### Atomic displacement parameters (Å^2^)

**Table:**

	*U*^11^	*U*^22^	*U*^33^	*U*^12^	*U*^13^	*U*^23^
O1	0.0699 (17)	0.0814 (19)	0.0879 (18)	0.0089 (14)	0.0514 (15)	−0.0122 (15)
O2	0.0556 (16)	0.101 (2)	0.0781 (17)	0.0107 (14)	0.0441 (14)	0.0021 (15)
O3	0.0572 (16)	0.107 (2)	0.0841 (18)	−0.0104 (14)	0.0462 (15)	0.0008 (15)
O4	0.0587 (16)	0.094 (2)	0.0815 (17)	−0.0156 (14)	0.0465 (14)	−0.0159 (14)
N1	0.0423 (17)	0.072 (2)	0.0512 (17)	0.0008 (14)	0.0278 (15)	−0.0056 (15)
N2	0.0450 (18)	0.073 (2)	0.0508 (17)	−0.0036 (15)	0.0279 (15)	0.0004 (15)
C1	0.054 (2)	0.064 (3)	0.063 (2)	0.0040 (19)	0.038 (2)	0.003 (2)
C2	0.051 (2)	0.058 (2)	0.056 (2)	−0.0022 (18)	0.0348 (19)	0.0027 (18)
C3	0.049 (2)	0.068 (3)	0.058 (2)	0.0026 (18)	0.029 (2)	0.007 (2)
C4	0.060 (2)	0.093 (3)	0.062 (3)	−0.010 (2)	0.030 (2)	0.003 (2)
C5	0.089 (3)	0.093 (3)	0.063 (3)	−0.020 (3)	0.050 (3)	−0.010 (2)
C6	0.076 (3)	0.082 (3)	0.060 (2)	0.001 (2)	0.047 (2)	−0.002 (2)
C7	0.048 (2)	0.067 (2)	0.048 (2)	0.0001 (18)	0.0316 (18)	0.0034 (18)
C8	0.049 (2)	0.067 (2)	0.059 (2)	0.0068 (19)	0.036 (2)	0.0113 (19)
C9	0.059 (2)	0.072 (3)	0.057 (2)	−0.0047 (19)	0.030 (2)	−0.001 (2)
C10	0.052 (2)	0.075 (3)	0.061 (2)	−0.008 (2)	0.036 (2)	−0.012 (2)
C11	0.054 (2)	0.067 (2)	0.049 (2)	−0.0031 (19)	0.029 (2)	−0.0094 (19)
C12	0.070 (3)	0.080 (3)	0.063 (3)	0.009 (2)	0.034 (2)	−0.008 (2)
C13	0.091 (3)	0.090 (3)	0.063 (3)	0.022 (3)	0.032 (3)	0.003 (2)
C14	0.135 (4)	0.070 (3)	0.077 (3)	0.010 (3)	0.065 (3)	0.001 (2)
C15	0.103 (3)	0.065 (3)	0.075 (3)	−0.010 (2)	0.060 (3)	−0.012 (2)
C16	0.069 (2)	0.057 (2)	0.051 (2)	−0.009 (2)	0.036 (2)	−0.0096 (19)
C17	0.063 (2)	0.059 (2)	0.060 (2)	−0.014 (2)	0.041 (2)	−0.0183 (19)

### Geometric parameters (Å, º)

**Table:**

O1—C1	1.201 (4)	C5—H5A	0.9300
O2—C8	1.207 (3)	C6—C7	1.365 (4)
O3—C10	1.195 (4)	C6—H6A	0.9300
O4—C17	1.203 (4)	C7—C8	1.476 (4)
N1—C8	1.381 (4)	C9—H9A	0.9700
N1—C1	1.391 (4)	C9—H9B	0.9700
N1—C9	1.425 (4)	C10—C11	1.480 (5)
N2—C17	1.386 (4)	C11—C12	1.369 (4)
N2—C10	1.389 (4)	C11—C16	1.372 (4)
N2—C9	1.441 (4)	C12—C13	1.380 (5)
C1—C2	1.473 (4)	C12—H12A	0.9300
C2—C3	1.362 (4)	C13—C14	1.363 (5)
C2—C7	1.366 (4)	C13—H13A	0.9300
C3—C4	1.364 (5)	C14—C15	1.382 (5)
C3—H3A	0.9300	C14—H14A	0.9300
C4—C5	1.369 (5)	C15—C16	1.358 (5)
C4—H4A	0.9300	C15—H15A	0.9300
C5—C6	1.394 (5)	C16—C17	1.470 (5)
			
C8—N1—C1	111.6 (3)	N1—C9—N2	113.7 (3)
C8—N1—C9	124.3 (3)	N1—C9—H9A	108.8
C1—N1—C9	123.8 (3)	N2—C9—H9A	108.8
C17—N2—C10	111.8 (3)	N1—C9—H9B	108.8
C17—N2—C9	125.1 (3)	N2—C9—H9B	108.8
C10—N2—C9	122.9 (3)	H9A—C9—H9B	107.7
O1—C1—N1	124.6 (3)	O3—C10—N2	125.0 (4)
O1—C1—C2	130.0 (3)	O3—C10—C11	129.2 (3)
N1—C1—C2	105.4 (3)	N2—C10—C11	105.8 (3)
C3—C2—C7	122.1 (3)	C12—C11—C16	121.5 (4)
C3—C2—C1	129.0 (3)	C12—C11—C10	130.7 (4)
C7—C2—C1	108.9 (3)	C16—C11—C10	107.8 (3)
C2—C3—C4	117.3 (3)	C11—C12—C13	117.0 (4)
C2—C3—H3A	121.4	C11—C12—H12A	121.5
C4—C3—H3A	121.4	C13—C12—H12A	121.5
C3—C4—C5	121.3 (3)	C14—C13—C12	121.6 (4)
C3—C4—H4A	119.4	C14—C13—H13A	119.2
C5—C4—H4A	119.4	C12—C13—H13A	119.2
C4—C5—C6	121.5 (4)	C13—C14—C15	120.8 (4)
C4—C5—H5A	119.3	C13—C14—H14A	119.6
C6—C5—H5A	119.3	C15—C14—H14A	119.6
C7—C6—C5	116.2 (3)	C16—C15—C14	117.7 (4)
C7—C6—H6A	121.9	C16—C15—H15A	121.1
C5—C6—H6A	121.9	C14—C15—H15A	121.1
C6—C7—C2	121.6 (3)	C15—C16—C11	121.4 (4)
C6—C7—C8	130.6 (3)	C15—C16—C17	129.8 (4)
C2—C7—C8	107.7 (3)	C11—C16—C17	108.8 (3)
O2—C8—N1	124.1 (3)	O4—C17—N2	124.6 (3)
O2—C8—C7	129.7 (3)	O4—C17—C16	129.6 (4)
N1—C8—C7	106.2 (3)	N2—C17—C16	105.8 (3)
			
C8—N1—C1—O1	−177.0 (3)	C17—N2—C9—N1	−67.9 (4)
C9—N1—C1—O1	−3.1 (5)	C10—N2—C9—N1	117.6 (3)
C8—N1—C1—C2	2.8 (3)	C17—N2—C10—O3	178.7 (3)
C9—N1—C1—C2	176.7 (3)	C9—N2—C10—O3	−6.2 (5)
O1—C1—C2—C3	−1.7 (6)	C17—N2—C10—C11	−1.4 (3)
N1—C1—C2—C3	178.5 (3)	C9—N2—C10—C11	173.8 (3)
O1—C1—C2—C7	179.8 (4)	O3—C10—C11—C12	2.6 (6)
N1—C1—C2—C7	0.0 (3)	N2—C10—C11—C12	−177.4 (3)
C7—C2—C3—C4	2.1 (5)	O3—C10—C11—C16	−177.6 (4)
C1—C2—C3—C4	−176.2 (3)	N2—C10—C11—C16	2.5 (4)
C2—C3—C4—C5	−0.7 (5)	C16—C11—C12—C13	0.0 (5)
C3—C4—C5—C6	−1.2 (6)	C10—C11—C12—C13	179.8 (3)
C4—C5—C6—C7	1.7 (5)	C11—C12—C13—C14	0.7 (6)
C5—C6—C7—C2	−0.3 (5)	C12—C13—C14—C15	−1.0 (6)
C5—C6—C7—C8	179.1 (3)	C13—C14—C15—C16	0.5 (6)
C3—C2—C7—C6	−1.6 (5)	C14—C15—C16—C11	0.3 (5)
C1—C2—C7—C6	177.0 (3)	C14—C15—C16—C17	−177.0 (3)
C3—C2—C7—C8	178.8 (3)	C12—C11—C16—C15	−0.6 (5)
C1—C2—C7—C8	−2.5 (3)	C10—C11—C16—C15	179.6 (3)
C1—N1—C8—O2	175.4 (3)	C12—C11—C16—C17	177.2 (3)
C9—N1—C8—O2	1.5 (5)	C10—C11—C16—C17	−2.6 (4)
C1—N1—C8—C7	−4.3 (3)	C10—N2—C17—O4	179.2 (3)
C9—N1—C8—C7	−178.1 (3)	C9—N2—C17—O4	4.2 (5)
C6—C7—C8—O2	5.1 (6)	C10—N2—C17—C16	−0.2 (3)
C2—C7—C8—O2	−175.5 (3)	C9—N2—C17—C16	−175.2 (3)
C6—C7—C8—N1	−175.3 (3)	C15—C16—C17—O4	0.0 (6)
C2—C7—C8—N1	4.1 (3)	C11—C16—C17—O4	−177.6 (3)
C8—N1—C9—N2	−69.4 (4)	C15—C16—C17—N2	179.3 (3)
C1—N1—C9—N2	117.4 (3)	C11—C16—C17—N2	1.8 (4)

### Hydrogen-bond geometry (Å, º)

**Table:**

*D*—H···*A*	*D*—H	H···*A*	*D*···*A*	*D*—H···*A*
C3—H3*A*···O3^i^	0.93	2.43	3.150 (6)	134
C4—H4*A*···O4^ii^	0.93	2.60	3.300 (5)	133
C15—H15*A*···O2^iii^	0.93	2.46	3.271 (7)	146

Symmetry codes: (i) *x*−1/2, −*y*+3/2, *z*−1/2; (ii) −*x*, −*y*+1, −*z*; (iii) *x*, −*y*+1, *z*+1/2.
